# Metabolic crosstalk between membrane and storage lipids facilitates heat stress management in *Schizosaccharomyces pombe*

**DOI:** 10.1371/journal.pone.0173739

**Published:** 2017-03-10

**Authors:** Mária Péter, Attila Glatz, Péter Gudmann, Imre Gombos, Zsolt Török, Ibolya Horváth, László Vígh, Gábor Balogh

**Affiliations:** Institute of Biochemistry, Biological Research Centre, Hungarian Academy of Sciences, Szeged, Hungary; University of Cambridge, UNITED KINGDOM

## Abstract

Cell membranes actively participate in stress sensing and signalling. Here we present the first in-depth lipidomic analysis to characterize alterations in the fission yeast *Schizosaccharomyces pombe* in response to mild heat stress (HS). The lipidome was assessed by a simple one-step methanolic extraction. Genetic manipulations that altered triglyceride (TG) content in the absence or presence of HS gave rise to distinct lipidomic fingerprints for *S*. *pombe*. Cells unable to produce TG demonstrated long-lasting growth arrest and enhanced signalling lipid generation. Our results reveal that metabolic crosstalk between membrane and storage lipids facilitates homeostatic maintenance of the membrane physical/chemical state that resists negative effects on cell growth and viability in response to HS. We propose a novel stress adaptation mechanism in which heat-induced TG synthesis contributes to membrane rigidization by accommodating unsaturated fatty acids of structural lipids, enabling their replacement by newly synthesized saturated fatty acids.

## Introduction

Organisms have evolved a variety of homeostatic strategies to cope with stress due to abrupt environmental fluctuations. Cellular stress responses are graded and appear to be proportional to the severity of applied stress [1]. Severe stress causes macromolecular damage [2]. However, small environmental changes that stress cells do not detectably impair cellular infrastructure and are able to induce homeostatic mechanisms that readjust cellular physiology [1,3].

One of the most important and widely studied environmental stressors is temperature fluctuation [4]. The heat stress response (HSR) is initiated by membrane-localised temperature sensors [5–7] that cause signal transduction events to alter transcriptional, translational and post-translational control processes. It was previously demonstrated that a subset of heat stress (HS)-induced thermoprotectants, including heat shock proteins and trehalose, act as membrane-stabilizing factors [8–13]. The key consequences of membrane lipid-controlled sensing and signalling of HS include membrane lipid rearrangement, signalling lipid generation via activation of various lipases and *de novo* synthesis of mediator-type sphingolipids and have been recently reviewed for various yeast, plant and mammalian species [3,14–18].

In addition to structural and signalling lipids, the participation of storage lipids in stress response has also been highlighted in the literature. Triglycerides (TGs) and steryl esters (SE) accumulate intracellularly in specialized storage structures called lipid droplets (LDs) that are dynamic organelles serving as hubs of lipid metabolism and energy homeostasis [19–23]. They serve as metabolic stores for generating chemical energy, membrane components and signal mediating lipids, and LD dysfunction is associated with many diseases [20]. It is now apparent that LDs participate in much broader roles than previously thought. For example, accumulating evidence points to their importance in inflammation [24] and in neurodegeneration [25,26]. With respect to stress response, the synthesis of TG and concomitant LD biogenesis was reported to be a hallmark of mammalian cellular stress response [24,27,28] and stress response in plants [29]. In *Saccharomyces cerevisiae* (*S*. *cerevisiae*), ER stress has been shown to be associated with enhanced LD formation [30]. Furthermore, treatment with the TOR kinase inhibitor rapamycin results in rapid LD replenishment in this yeast. Induction of LD formation was shown to be caused by increased TG, but not SE, synthesis [31]. However, the role of TG synthesis in stress-induced lipid metabolic reprogramming remains to be elucidated.

The budding yeast *S*. *cerevisiae* and the fission yeast *Schizosaccharomyces pombe (S*. *pombe)* are popular eukaryotic model systems for studying basic biological processes in attempt to understand homologous processes in more complex organisms such as mammals. In both yeasts, the terminal step of TG synthesis proceeds by two different mechanisms, one governed by acyl-CoA-dependent acyltransferase Dga1p in both yeast species and the second by acyl-CoA-independent phospholipid-diacylglycerol acyltransferases Lro1p in *S*. *cerevisiae* and Plh1p in *S*. *pombe* [32–34].

Based on the above findings, we aimed to systematically identify alterations in the lipidome of wild-type (WT) and TG-deficient *S*. *pombe* strains exposed to mild HS. Currently, mass spectrometry (MS)-based lipidomic methods have been developed to the point that their usefulness as an “omics”-scale tool in various fields of biology have been soundly established [35]. In this study, we made several improvements in the lipidomics workflow that included rapid disruption of fission yeast cells, simple one-phase lipid extraction and high-throughput comprehensive shotgun MS profiling that affords rapid quantitation of both polar and neutral lipids. Most importantly, our results demonstrate that preferentially upregulated TG synthesis is an active HS-inducible process that efficiently serves to maintain membrane homeostasis and, thereby, highlights the role of metabolic crosstalk between membrane and storage lipids during HSR.

## Materials and methods

### Materials

Lipid standards were from Avanti Polar Lipids (Alabaster, AL, USA). Solvents for extraction and MS analyses were liquid chromatographic grade (Merck, Darmstadt, Germany) and Optima LC–MS grade (Thermo Fisher Scientific, Waltham, MA, USA). All other chemicals were the best available grade purchased from Sigma-Aldrich (Steinheim, Germany).

### Yeast strains and growth conditions

*S*. *pombe* strains used in this study are listed in S1 Table. BRC1 [36] was used to generate *dga1Δ*, *plh1Δ* and *dga1Δplh1Δ* (DKO) strains. Mutants were constructed according to the PCR method described by Krawchuk and Wahls [37]. For constructing the null mutants, pBSK(−) plasmid (Stratagene) containing the 1.8-kb ura4+ fragment of pREP41 (kindly provided by S. Forsburg) was used as template. The DKO strain was obtained by genetic crossing of the two single-mutant strains. Primers are listed in S1 Table. Transformations were carried out according to a standard protocol [38]. Variant strain mutations were confirmed by PCR.

Cells were grown at 30°C in EMM prepared according to [39], and supplemented with leucine (1.71 mM) and uracil (2.01 mM). Cells in exponential phase (3–5×10^6^ cells/mL) were used for all experiments.

### Viability staining of *S*. *pombe* cells

Trypan blue and methylene blue were used for estimating the ratio of live to dead cells [40,41]. Both dyes selectively coloured dead cells blue. Trypan blue uptake indicates the damage of the cell membrane [42], while methylene blue staining reports about metabolic disorders, *e*.*g*., dead cells are unable to reduce the dye [43].

Control and heat-treated *S*. *pombe* cultures were stained by adding equal volumes of dyes (0.4 w/v% trypan blue or 0.1 w/v% methylene blue in PBS) to 250 μL cultures. After 5 min of staining, cells were collected by centrifugation (3 min at 800 xg). 450 μL of supernatant was discarded and cells were resuspended in the remaining ~50 μL solutions and analysed by light microscopy.

### Determination of thermosensitivity and *de novo* protein synthesis

Cells were subjected to HS at 40°C for 1 h. Samples were then serially diluted (10x) and 10 μL aliquots were spotted onto YES plates and incubated at 30°C for 1 to 4 days. To determine the growth rates of the different strains after HS, liquid cultures were allowed to recover at 30°C and samples were taken at various time intervals for OD_600nm_ absorbance spectroscopic measurements (WPA CO8000, Biochrom Ltd., Cambridge, UK). In choline supplemenation experiments, 1 mM choline was added to the culture during HS.

*De novo* protein synthesis was measured by adding 20 μL of ^14^C protein hydrolysate (Amersham CFB25, 50 μCi/mL) to 2 mL cell suspension for 1 h before lysis. After brief centrifugation, cells were resuspended in 250 mM NaOH and disrupted using a Bullet Blender Gold homogenizer (Next Advance, Inc., Averill Park, NY, USA) in the presence of zirconium oxide beads (0.5 mm) at speed 8 for 3 min at 4°C. The homogenate was centrifuged (1000 x*g*, 4°C, 3 min) and proteins were precipitated on ice by mixing samples with an additional 1/4 sample volume of 50% (v/v) aqueous TCA for 20 min. After centrifugation (10,000 x*g*, 4°C, 10 min), pellets were washed twice with 1 mL −20°C acetone and solubilized in 1x protein loading buffer for 3 h. Proteins were separated on 10% SDS-PAGE and prepared for fluorography as in [44].

### One-step methanolic lipid extraction

After growing cultures according to various treatment protocols, 10^8^ cells were harvested by filtration. Centrifugation was avoided because it has been shown to cause MAPK activation that affects stress signalling pathways [45]. Cells were disrupted in water using a bullet blender homogenizer in the presence of zirconium oxide beads (0.5 mm) at speed 8 for 3 min at 4°C. Protein concentration of cell homogenates was determined using the Micro BCA™ Protein Assay Kit (Thermo Fisher Scientific, Waltham, MA, USA). A portion (40 μL of 500 μL total volume) of the homogenate was immediately subjected to a simple one-phase methanolic (MeOH) lipid extraction. First, the homogenate was sonicated in 1 mL MeOH containing 0.001% butylated hydroxytoluene (as antioxidant) in a bath sonicator for 5 min, then shaken for 5 min, centrifuged at 10,000 x*g* for 5 min. The supernatant was transferred into a new Eppendorf tube and stored until MS measurement (~40 nmol total lipid/mL MeOH). Validation of MeOH extraction was performed as described in S1 Appendix.

### Mass spectrometry

ESI-MS analyses were performed using a LTQ-Orbitrap Elite instrument (Thermo Fisher Scientific, Bremen, Germany) equipped with a TriVersa NanoMate robotic nanoflow ion source (Advion BioSciences, Ithaca, NY), using chips with 5.5 μm diameter spraying nozzles. The ion source was controlled by Chipsoft 8.3.1 software. The ionization voltages were +1.3 kV and −1.9 kV in positive and negative modes, respectively, and back-pressure 1 psi for both modes. The temperature of the ion transfer capillary was 330°C. Acquisitions were performed at the mass resolution R_*m/z* 400_ = 240,000.

The lipid classes phosphatidylcholine (PC), lysophosphatidylcholine (LPC), diacylglycerol (DG), triacylglycerol (TG) and ergosteryl ester (EE) were detected and quantified using the positive ion mode, while phosphatidylethanolamine (PE), monomethyl-phosphatidylethanolamine (MMPE), dimethyl-phosphatidylethanolamine (DMPE), phosphatidylinositol (PI), phosphatidylserine (PS), the lyso derivatives LPE, LPI, LPS, phosphatidic acid (PA), phosphatidylglycerol (PG), cardiolipin (CL), ceramide (Cer), inositolphosphoceramide (IPC) and mannosyl-inositolphosphoceramide (MIPC) were detected and quantified using the negative ion mode. Lipids were identified by LipidXplorer software [46] by matching the m/z values of their monoisotopic peaks to the corresponding elemental composition constraints. The mass tolerance was 3 ppm. Further details of MS measurements are presented in S2 Appendix.

Double bond index/sat values (DBI/sat) were calculated for each major membrane lipid classes as follows:

DBI/sat = Σ(db x [L_i_]) / Σ(db_0_ x [L_i_]), where db is the total number of double bonds in a given lipid species L_i_, db_0_ is the total number of saturated fatty acyl chains in the same lipid species L_i_, and the square bracket indicates the mol% of lipid species L_i_ in the specified lipid class (*e*.*g*., in PC).

### Lipid species annotation

Lipid classes and species were annotated according to the classification systems for lipids [47,48]. For glycerolipids, the “Lipid class(FA1_FA2)” format specifies the structures of the fatty acyl (FA) side chains (*e*.*g*., PC(10:0_18:1), whereas the “Lipid class(sn1/sn2)” format specifies the side chain regiochemistry, too (*e*.*g*., PC(16:0/18:1)). In sum formulas, *e*.*g*., PE(36:2), the total numbers of carbons followed by double bonds for all chains are indicated. For sphingolipids, first the number of hydroxyl groups in the long chain base (*e*.*g*., “t” for the three hydroxyls of phytosphingosine), the number of carbon atoms and then the number of double bonds are indicated followed by the *N*-acyl chain composition, such as Cer(t20:0/24:0). The sum formula, *e*.*g*., Cer(44:0:3), specifies first the total number of carbons in the long chain base and FA moiety then the sum of double bonds in the long chain base and the FA moiety followed by the sum of hydroxyl groups in the long chain base and the FA moiety.

### Statistics

Lipidomic results are presented as mean ± SD; significance was determined according to Storey and Tibshirani [49] and was accepted for *p*<0.05 corresponding to a false discovery rate <0.035. Cluster analyses of lipidomic datasets were performed using MetaboAnalyst [50].

## Results and discussion

### Long-lasting growth arrest due to HS in the absence of TG synthesis

**Fig 1:**
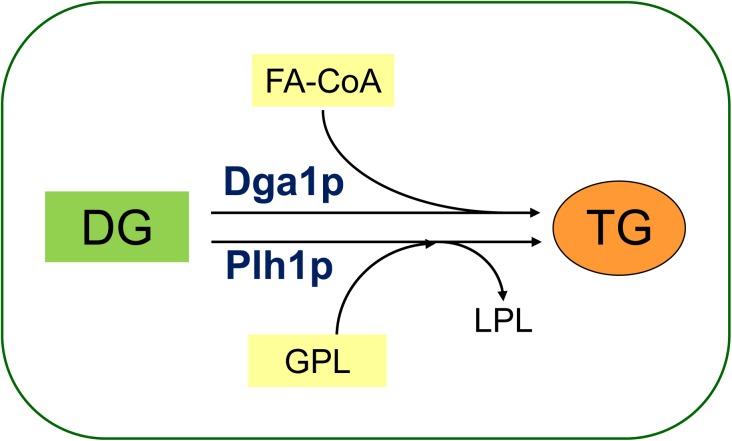
Schematic representation of TG synthesis in *S*. *pombe*. Dga1p uses FA-CoA and diacylglycerol (DG) to produce TG, while Plh1p catalyses the direct FA transfer between a glycerophospholipid (GPL) and DG to generate TG and a lysophospholipid (LPL).

**Fig 2:**
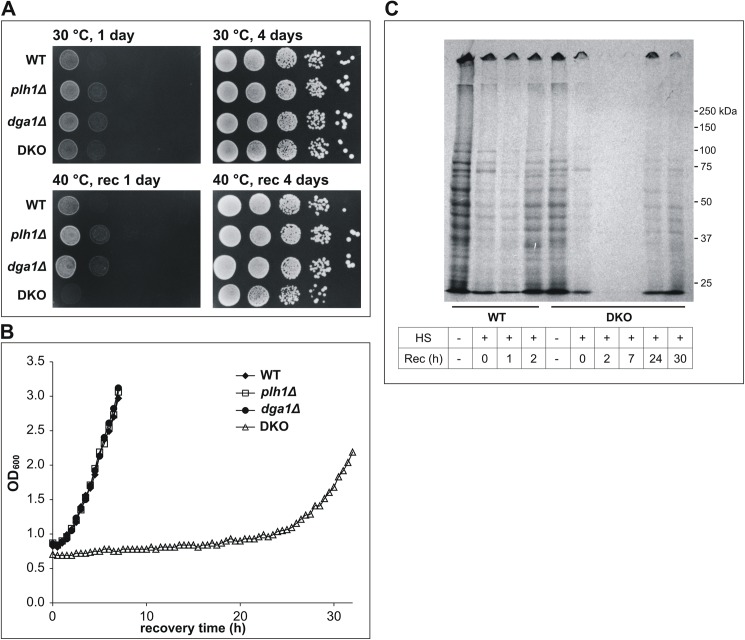
Severe growth retardation and long translational inhibition in heat-stressed DKO cells. WT and TG-deficient *S*. *pombe* cells were either untreated (30°C) or exposed to HS (40°C) for 1 h. Cells were allowed to recover (rec) at 30°C, and detection of thermal resistance was performed by (A) spot tests on agar plates after 1 and 4 days, and by (B) recording growth curves for liquid cultures (results for one of three independent replicate experiments are shown). (C) Fluorogram showing de novo protein synthesis by ^14^C-labelling (WT and DKO stains are shown).

To maintain controlled lipid metabolic conditions, we grew all yeast cells for this study on minimal media to mid-log phase at 30°C. Since we intended to explore the role that TG may play in HSR in *S*. *pombe*, mutants were first generated in which one (*dga1Δ* or *plh1Δ*) or both (*dga1Δplh1Δ*, DKO) genes that encode TG synthesizing enzymes were deleted. Dga1p is an acyl-CoA-dependent diacylglycerol O-acyltransferase that catalyses the reaction between FA-CoA and diacylglycerol (DG) to produce TG (and CoA), while Plh1p is a phospholipid-diacylglycerol acyltransferase that catalyses the direct FA transfer between a glycerophospholipid (GPL) and DG in an acyl-CoA-independent manner to generate TG and a lysophospholipid (LPL) (Fig 1). In agreement with the observations of Zhang *et al*. [32], the mutants did not show apparent morphological or growth defects (Fig 2A, upper plates).

Next, we tested the stress resistance of the deletion strains, compared to that of the WT, exposed to HS for 1 h at 40°C. This condition is considered to be mild HS because, at this temperature, WT *S*. *pombe* cells do not grow and divide, but are quantitatively viable [11,51]. The mutant strains also displayed 100% viability immediately after HS as assessed by either methylene blue or trypan blue staining (S1A Fig). However, the spot tests developed on agar plates revealed a striking difference in colony densities after 1 day recovery at 30°C; DKO cells hardly grew, while colonies of the other strains became larger (Fig 2A, lower left plate). After 4 days recovery, no difference was seen in the colony-forming abilities for any of the mutant strains compared to WT (Fig 2A, lower right plate). Growth curves, recorded in liquid culture after HS, further confirmed the prolonged transient growth arrest of heat-stressed DKO cells. Fig 2B shows that there were no differences between the growth reinitiation of the single knockout variants and that for the WT cells. However, the DKO strain began to grow only after an extremely long (~24 h) delay compared to the other strains. Importantly, viability staining at various time points during the lag phase unambiguously excluded the possibility that the DKO cell growth arrest was due to cell death since the proportion of viable cells was ≥90% even after 22 h recovery time (S1B Fig).

To investigate whether growth arrest in DKO cells is coupled with translational inhibition, we assessed *de novo* protein synthesis by ^14^C-labelling. For WT cells, newly synthesized proteins appeared after 2 h recovery at 30°C. However, in the DKO strain, new protein synthesis was apparent only after ~24 h (Fig 2C). Translational arrest is a general cell survival strategy as a part of the integrated stress response that allows diversion of cellular resources to ensure cell survival [16,52,53].

### Development of a simple, fast method for the analysis of membrane and storage lipids

The dynamic changes in cellular lipidomes during stress response are expected to occur over relatively wide ranges of time and concentration, and may affect structure and polarity changes for potentially many lipid types. Although several powerful methods exist for MS lipidomic analysis of yeast cells [54–57], we introduced several improvements to meet desired goals of rapid high-throughput, extensive coverage, and improved safety. Our lipidomic workflow included the collection of cell pellet via rapid filtering, cell lysis using bullet blender homogenization, easy one-step MeOH extraction, which eliminates the harmful exposure to chloroform, and comprehensive shotgun ESI-MS(/MS) assessment of extracted lipids using an Orbitrap Elite mass spectrometer.

Lipidomic analyses were performed on both unstressed and heat-stressed lipid extracts. We identified and quantified ~130 molecular species encompassing 19 lipid classes. In S2 Table, both qualitative and quantitative lipid data are summarized. Qualitative data (mol% values for membrane lipids; ML = GPL + sphingolipids (SL) + DG) report on the physical state of the membrane, while lipid/protein (nmol/mg) values are quantitative measures reporting on metabolic processes.

### Genetic manipulation of TG level alone, or in combination with applied HS, results in distinct lipidomic fingerprints

**Fig 3:**
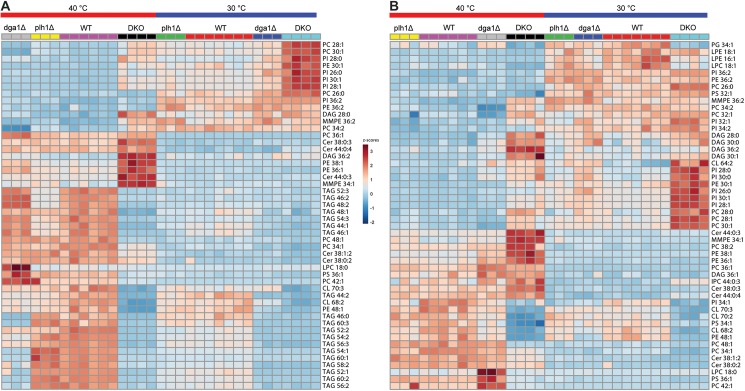
Unsupervised statistics reveal separation of lipidomes due to deletion of TG synthesis genes or HS. Heatmap representation of hierarchical clustering (A) with or (B) without storage lipids; top 50 most significant species were selected based on ANOVA, Euclidean distance, clustering algorithm Ward; heat color code represents normalized values (z-scores) that range between [−2] (darkest shade of blue) and [+3] (darkest shade of red). Three to seven independent experiments are shown for control and HS-treated (40°C, 1 h) cells.

Lipidomic MS results were analysed by comparing relative amounts of the various molecular species for the four individual sample strains (WT, *dga1Δ*, *plh1Δ*, DKO), for which more than 700 statistically-significant distinctions including all possible pairwise comparisons (*p*<0.05; S2 Table) were observed. The heatmap representation of hierarchical cluster analysis results illustrates distinctively different patterns for all experimental conditions (Fig 3A). From these results we conclude that gene deletion alone, or in combination with HS treatment, gives rise to distinctly different *S*. *pombe* lipidomic fingerprints. Importantly, the lipidomes of the four strains separated without considering TG (and ergosteryl ester (EE)), *i*.*e*., based merely on membrane lipids at both 30 and 40°C (Fig 3B). These results show that storage lipid (especially TG) metabolism has a clear effect on membrane lipid composition under both unstressed and stressed conditions.

### TG synthesis has priority during HS in *S*. *pombe*

**Fig 4:**
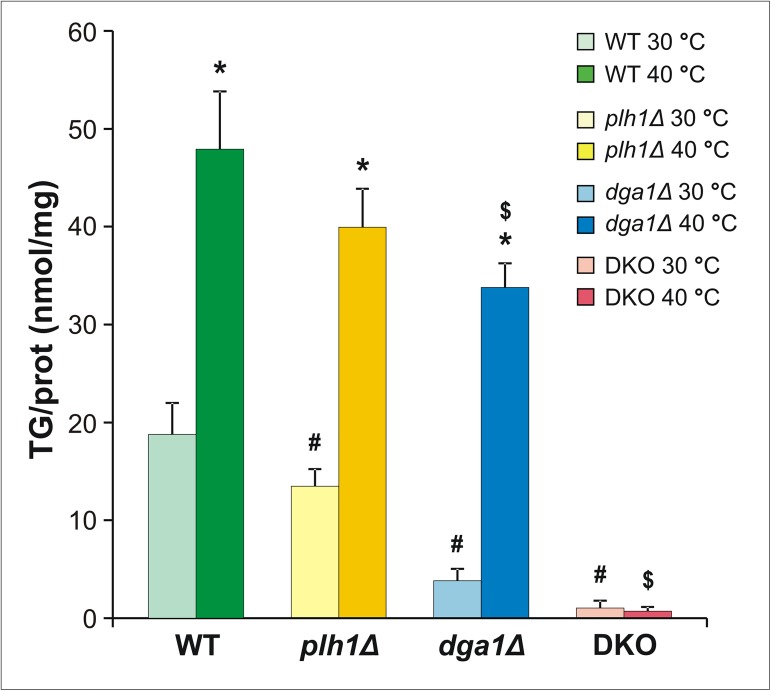
TG synthesis has priority during HS in *S*. *pombe*. Cells were untreated (30°C) or stressed at 40°C for 1 h. Changes in TG levels due to gene deletion and/or HS are shown. Data are expressed as TG/protein (nmol/mg) and shown as mean ± SD, n = 3 for *plh1Δ* and *dga1Δ*, n = 4 for DKO, and n = 7 for WT; * p<0.05 (30°C vs 40°C), # p<0.05 (WT vs mutants at 30°C), $ p<0.05 (WT vs mutants at 40°C).

Quantitative lipidomics results revealed that all mutants showed significantly reduced TG levels in comparison to that for WT cells at 30°C (Fig 4). In the DKO strain, the cellular TG mass dropped to a minimal basal value (~95% loss compared to WT). Among the single-mutant strains, a smaller decrease was detected for *plh1Δ* cells (~30% loss), whereas in *dga1Δ* cells the reduction was much larger (~80% loss). We conclude that the two genes are not redundant. The results were different in response to HS, where TG increased by a very significant 2.6-fold for WT cells (Fig 4). This was the largest detected change among all lipid classes (S2 Table) in spite of the fact that neither *dga1* nor *plh1* were previously reported to be transcriptionally activated due to HS by DNA microarray analysis [58]. The *plh1Δ* mutant displayed very similar, ~3-fold TG elevation, whereas in the more TG-deficient *dga1Δ* mutant we detected a much larger, ~9-fold enhancement compared to the unstressed level for which the TG content closely approached the value found for *plh1Δ*. Since the absolute TG content of the heat-stressed single-mutant strains reached higher values than expected (*i*.*e*., only ~15–30% below the WT value), our data show that as long as the cells possess remaining TG synthesis activity, homeostatic mechanisms are able to sustain increased TG synthesis. This suggests that *S*. *pombe* cells give high priority to elevating TG levels during HS.

### Heat sensitivity of DKO cells correlates with increased signalling lipid generation relative to that for WT cells

Quantitative results highlighted several significant changes in signalling lipids such as DG, PA, Cer and IPC (S2 Table). The most prominent differences were for Cer and DG, which are centrally involved in glycerolipid and sphingolipid biosynthesis, respectively (S2 Fig), and that possess versatile signalling roles.

**Fig 5:**
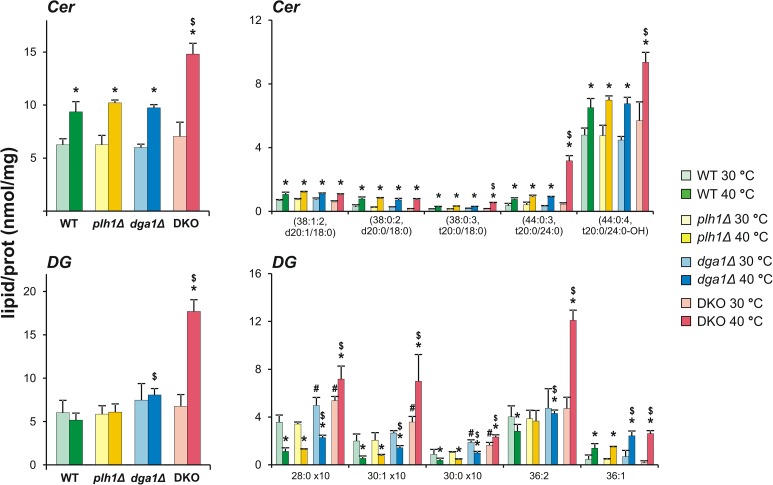
Growth arrest of heat-stressed DKO cells correlates with enhanced signalling lipid generation. Changes in the amounts of Cer and DG by lipid class and species levels are shown. Cells were untreated (30°C) or stressed at 40°C for 1 h. Values are expressed as mean ± SD of lipid/protein values (nmol/mg), n = 3 for *plh1Δ* and *dga1Δ*, n = 4 for DKO, and n = 7 for WT; * p<0.05 (30°C vs 40°C), # p<0.05 (WT vs mutants at 30°C), $ p<0.05 (WT vs mutants at 40°C).

The 1.4-2-fold accumulation of Cer in all strains studied is considered to be a common characteristic of the HSR (Fig 5). Results of previous studies demonstrated that regulation of acute activation of the *de novo* SL pathway in response to HS is evolutionary conserved among yeast and human cells [14,15,59,60]. Recently, it was shown that Ypk1, a TORC2-dependent protein kinase, controls the phosphorylation of ceramide synthase and increases its specific activity upon HS [61]. The rapid increase in SLs was proposed to trigger changes in several cellular processes including transcription, translation, growth and actin organization [16]. Notably, besides the dominant contribution of the main hydroxy-phytoceramide (Cer(44:0:4, t20:0/24:0-OH); 1.4-fold elevation) to the total Cer increase, lipidomic data also revealed a distinguishing elevation for its biosynthetic precursor (Cer(44:0:3, t20:0/24:0); 7-fold elevation) in DKO cells (Fig 5).

DG also plays a crucial role in cell growth and development with direct activation of PKC being one of the most important DG-mediated responses [62,63]. Recently, it was suggested that the fundamental differences between the mitotic strategies of two fission yeast species, *S*. *pombe* and *S*. *japonicus*, might depend on divergence of the regulatory networks controlling lipin (PA-phosphohydrolase) activity to produce DG in a spatially restricted manner [64]. In the present study, DG level was found to be strictly controlled in *S*. *pombe* cells. In comparison to WT cells at 30°C, DG level remained unaltered in the unstressed single-mutant and DKO cells (Fig 5), where accumulation of the substrate would be reasonably expected due to deletion of TG synthesis genes. In addition, despite the elevation of TG levels, DG levels did not change in the WT or single-mutants under HS conditions. Only the double knockout in combination with HS (*i*.*e*., DKO cells exposed to 40°C) caused a metabolic imbalance that resulted in a very pronounced 2.6-fold increase in DG level (Fig 5). Moreover, in Fig 5 we show that underlying alterations in the amounts of DG are strongly species-dependent in all strains. The shorter chain DGs were significantly downregulated for the WT and single-mutants, but elevated for DKO cells. The 36:1 species were elevated in all strains, while the most abundant lipid species, 36:2, was upregulated 2.6-fold only in the double mutant. We conclude that, in the absence of TG synthesis, the enhanced generation of DG and Cer represents an overresponse in DKO cells, which could reasonably lead to the observed growth arrest and long-term protein translational inhibition in response to HS. Indeed, *in situ* choline supplementation during HS essentially suppressed the DG increase and shortened the growth retardation of DKO cells, conceivably due to the fact that choline induces the CDP-choline route of PC biosynthesis to consume DG (S3 Fig).

Differences between budding and fission yeast mutants that are unable to synthesize TG were previously established. The *dga1Δlro1Δ S*. *cerevisiae* mutant is viable and has no apparent growth defect in all growth phases [65]. In contrast, *S*. *pombe* DKO cells lose viability upon entry into stationary phase [32,66,67]. In these studies, the authors identified a surge in DG level (205% elevation for the main species DG 36:2) as a key lipid change that induced apoptosis, whereas Cer increase (35% for Cer 44:0:3) was claimed to evoke a non-apoptotic cell death program [66]. In the present study, we found a very similar change for DG 36:2, and an even larger elevation for Cer 44:0:3, in heat-stressed DKO cells, accompanied by a long, but transient, growth arrest phase. In fact, whether the increase in Cer leads to cell death or to viability is considered to be strongly organism- and growth condition-specific [3,15,68,69]. Likewise, the DG-activated isoforms of PKC can be either pro-apoptotic or anti-apoptotic [62,63,70,71]. Of note, it was reported that a marked increase in cellular DG content in the fission yeasts *S*. *japonicus* and *S*. *pombe*, both deficient in CDP-DG synthase function, coincided with a vastly decreased growth rate in chemically defined minimal medium at elevated temperature [72]. Similarly, the *S*. *cerevisiae* mutant that cannot synthesize neutral lipids is delayed at cell separation upon release from mitotic arrest and showed a marked increase of DG and PA [73].

### TG synthesis facilitates heat adaptation of membrane lipids

Tightly linked to the ability to respond to HS, the maintenance of physiological membrane fluidity by modulating lipid metabolic pathways is of critical importance for proper cell functioning, growth and viability, especially for poikilothermic organisms. This fundamental property is called homeoviscous adaptation [74] and involves many lipid-related elements. In response to HS, poikilotherms can decrease FA unsaturation, increase FA chain length or elevate the ratio of bilayer forming versus non-bilayer forming structural lipids [4,11,17,54,74–78].

**Fig 6:**
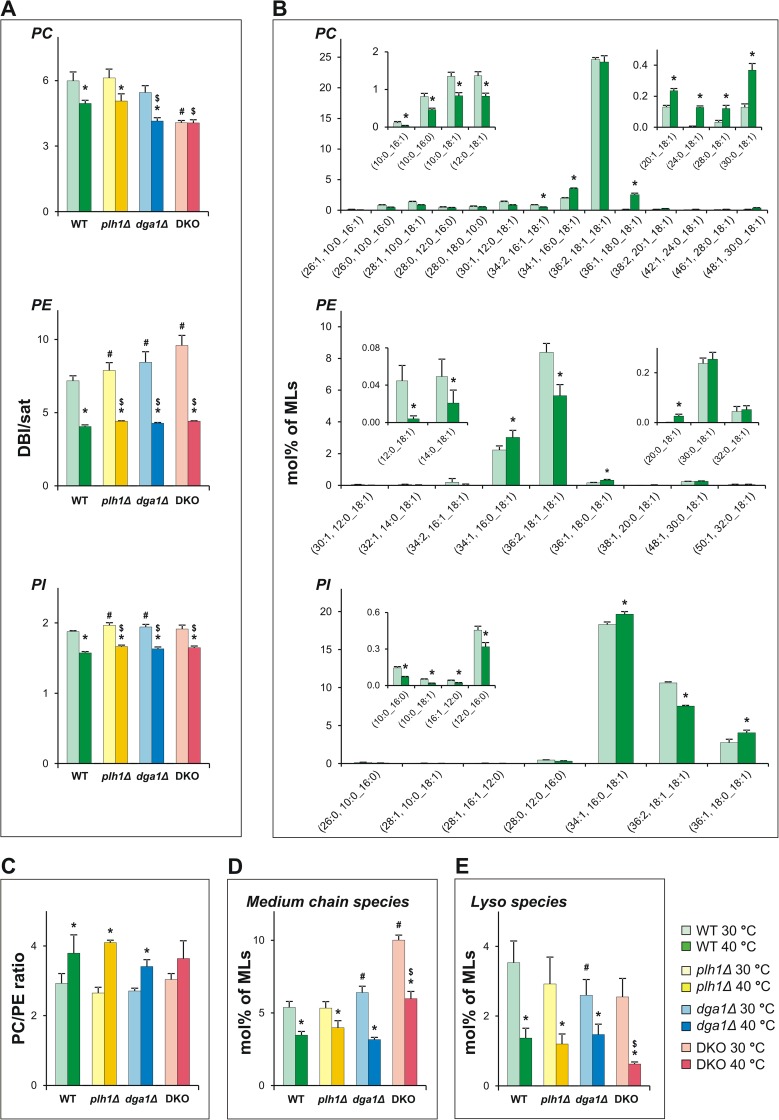
Changes in membrane lipid compositions. (A) Double bond index/saturated FA ratios (DBI/sat) for PC, PE and PI. (B) Lipid species compositional alterations for major membrane lipids PC, PE, and PI in the WT strain. (C) Changes in PC/PE ratios. (D) Changes in the contribution of medium chain FA-containing species to membrane lipid composition. (E) Alterations in lyso-lipid species. Cells were untreated (30°C) or stressed at 40°C for 1 h. Values are expressed as mol% of MLs (mean ± SD), n = 3 for *plh1Δ* and *dga1Δ*, n = 4 for DKO, and n = 7 for WT; * p<0.05 (30°C vs 40°C), # p<0.05 (WT vs mutants at 30°C), $ p<0.05 (WT vs mutants at 40°C). DBI/sat, double bond index/saturated FA ratio; PC, phosphatidylcholine; PE, phosphatidylethanolamine; PI, phosphatidylinositol.

Based on qualitative lipidomic data, we could identify several components of homeoviscous membrane adaptation that effectively function in the WT and single-mutant *S*. *pombe* strains. As a measure of membrane unsaturation, we calculated the double bond index/saturated FA ratios (DBI/sat [79]; for equation see Materials and Methods). Similar as for the budding yeast *S*. *cerevisiae* [54], the major GPLs in *S*. *pombe* are PC, PE and PI, which altogether account for ~75% of the ML fraction (S2 Table). We found significantly increased saturation (*i*.*e*., decreased DBI/sat ratios) as a result of HS for all major membrane lipid classes (Fig 6A). The increase of saturation occurred in a lipid class-dependent manner as indicated by alterations in the WT strain (Fig 6B). For PE and PI, the major diunsaturated species 36:2 (18:1/18:1) was substantially lowered by ~30%, whereas monounsaturated species 34:1 (16:0/18:1) and 36:1 (18:0/18:1) were significantly elevated. PC 36:2, which accounts for 75% of total PC, remained unchanged, while PC 36:1 was drastically increased (25- to 60-fold). In addition to the above abundant species, which are composed of long chain FAs (C16-C18), we identified medium chain (C10-C12) and very long chain (C20-C32) FA-containing components. The variation in the levels of these components served to increase the average FA chain length in two different ways in the heat-stressed samples; the medium components (*e*.*g*., PC(10:0_18:1) or PI(10:0_16:0)) were generally lowered, while the very long components (*e*.*g*., PC(30:0_18:1) or PE(32:0_18:1)) were elevated or not altered (Fig 6B). Furthermore, we observed significantly increased ratios of bilayer forming PC versus non-bilayer forming PE in the WT and single-mutant strains (~26–55% increase) (Fig 6C). In DKO cells, several of these changes in fluidity-altering lipids are also operational in response to HS, including increased saturation in PE and PI and the lowering of medium chain species (Fig 6A and 6D). Nevertheless, we identified several characteristics that reflect measurable differences in the membrane composition of DKO cells, such as the basally lower DBI/sat value for PC (that was not further lowered upon HS), the basally higher DBI/sat in PE, a defect in PC/PE increase, and the essentially higher contribution of medium chain species to membrane lipid composition at both temperatures (Fig 6A, 6C and 6D).

In addition to major structural lipids, significant changes occurred in LPLs, Cer and DG. The substantially reduced levels of LPLs with strong positive curvature-inducing and bilayer-destabilizing properties [80,81] could be identified for all strains (Fig 6E). With respect to membrane structural aspects, Cer and DG induce negative curvature to membranes due to their small polar headgroups [62,82]. Upon HS, the contribution of Cer increased from 3.7 to 7.3 mol%, while that of DG increased from 3.8 to 8.7 mol% for the membrane lipid fraction in DKO cells. These are sizeable and significantly larger (by ~200%) increases than measured for the other strains (S2 Table). We conclude that the above-described distinctive changes may unfavourably influence membrane composition and physical properties for the heat-challenged DKO mutant, whereas heat-induced modulation of TG synthesis for the WT and single-mutant strains likely supports dynamic changes in membrane properties aimed at maintaining cellular homeostasis.

### Changes in FA fluxes corroborate metabolic crosstalk between membrane and storage lipids

Previous reports concluded that storage lipid synthesis actively influences membrane lipid metabolism in various growth phases for both *S*. *cerevisiae* [83–86] and *S*. *pombe* [72] at various growth temperatures. To explore the nature of the proposed heat-induced TG-assisted membrane reorganization, we calculated how the whole cellular lipid content changed as a result of HS. Because the major reaction affected, *i*.*e*., TG synthesis, and also several other lipid metabolic reactions depend on FA transfer (S2 Fig), we analyzed the differences in FA fluxes expressed as FA/protein_(after HS–before HS)_ (nmol/mg/h) for GPL, SL, FFA, DG, TG and EE.

**Fig 7:**
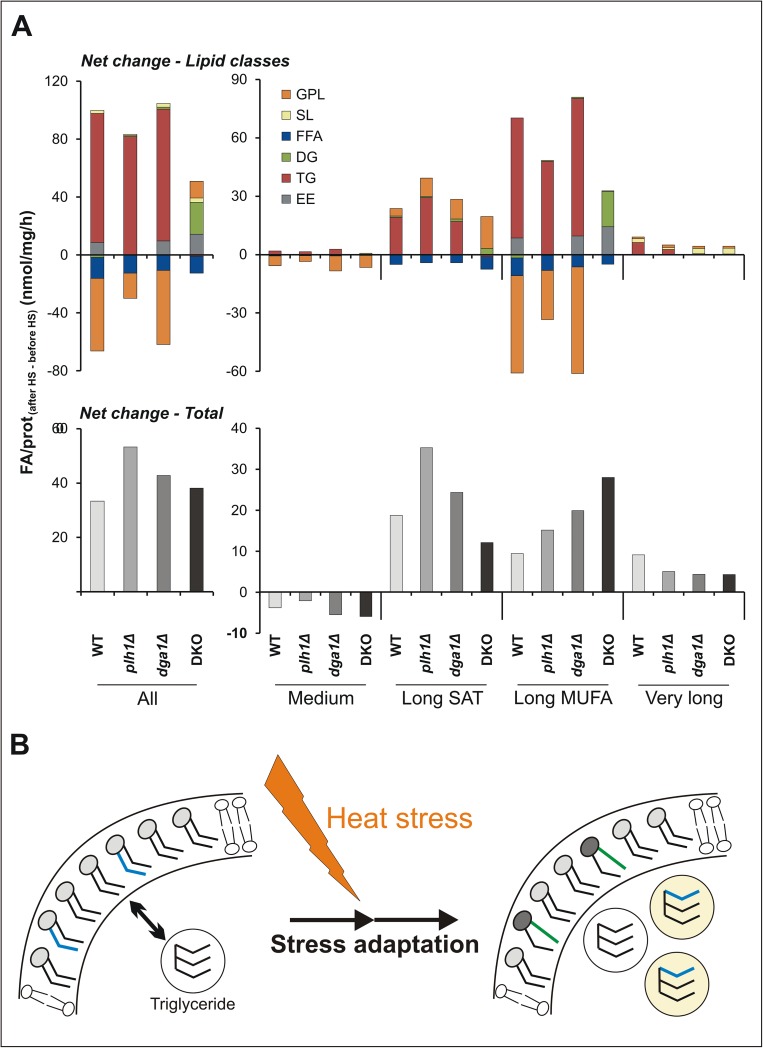
Changes in FA fluxes corroborate crosstalk between membrane and storage lipid metabolism. (A) Net changes expressed as FA/prot_(after HS–before HS)_ (nmol/mg/h) values for all FAs (upper panels) and for FA groups (medium– C10-C14, long SAT– 16:0 and 18:0, long MUFA– 16:1 and 18:1, very long C20-C32; lower panels). Average values are shown from n = 3 (for *plh1Δ* and *dga1Δ*), n = 4 (for DKO), and n = 7 (for WT) independent experiments. Data were reconstituted based on ESI-MS/MS fragmentation results with the exception of FFA, which was determined by GC-MS. (B) Graphical illustration of metabolic crosstalk between membrane and storage lipids.

Our analysis indicated that the net change in FA fluxes was positive for all strains (Fig 7A, lower left panel), *i*.*e*., that the quantity of synthesized FAs exceeded that of the consumed ones. As previously shown, the major lipid class with positive balance was TG for the WT and single-mutant strains, whereas the GPL and FFA fractions decreased (Fig 7A, upper left panel). For DKO cells, in the absence of TG formation, only the FFA fraction became depleted, but the balance was positive for GPL, DG and EE. Considering FA groups, our data revealed that the total net change was negative for the medium chain components, whereas it was positive for the long and very long chain groups in all strains (Fig 7A, lower right panel). The dominant changes occurred for long chain FAs. Among these, the saturated FAs were elevated in GPL (in all strains) and in TG (in the TG-containing strains) (Fig 7A, upper right panel) with the major contribution being 18:0 (S4 Fig). In contrast, monounsaturated FAs (MUFA; predominantly 18:1) were depleted from GPL and incorporated mainly into TG (and EE) in the TG-containing strains (Fig 7A and S4 Fig). In the *dga1Δ* mutant, 18:1 could be directly depleted by Plh1p, while in *plh1Δ*, phospholipase A/B actions may be responsible for its depletion from GPL. FA-CoAs as substrates for driving Dga1p enzymatic action can be derived directly from *de novo* FA synthesis or via activation of FFAs produced by various lipases. Taken together, these results strongly suggest that the depletion of 18:1 from GPL and its incorporation into TG are functionally interconnected processes and imply that the maintenance of proper membrane fluidity (*i*.*e*., rigidization in response to stress causing increased fluidization) might be most efficiently achieved via crosstalk between membrane and storage lipid metabolism (Fig 7B). Since *dga1* and *plh1* are transcriptionally inactive due to HS [58], it appears that such metabolic crosstalk might be regulated at the level of enzyme activities. It is highly conceivable, that the elevated temperature is sensed by Mga2, a transcription factor conserved among fungi, which was recently identified as a lipid-packing sensor in the ER membrane to control the FA synthesis and unsaturation [87].

In addition, LDs may also serve as a reservoir for rapidly supplying FAs for redefining membrane lipid composition after external stressors (*i*.*e*., non-physiological environmental conditions) are removed. A similar idea has been proposed for consideration in plant and microalgae research as a new approach to interpret the role of TG synthesis in response to different stressors, including HS [29]. The utilisation of TG in the post-stress state is presumably analogous to the process whereby cells exit the stationary phase, resume growth, and TG is hydrolysed and utilized for phospholipid synthesis [85]. Moreover, it should also be noted that the net decrease in GPL was largely overridden by the net TG mass produced (*i*.*e*., more TG formed than was necessary for facilitating membrane adaptation; Fig 7A, upper left panel). Expanding the putative versatile roles of LDs, we propose that this "extra" TG increase may also have an underlying rationale. Indeed, our results are in conformance with the previously proposed holdase chaperone function (*i*.*e*., that the HS-induced LD accumulation itself may serve as a reservoir for sequestering unfolded proteins during stress) [20,88]. In parallel, the concomitant translocation of the heat shock protein Hsp70 to the LD surface might be involved in chaperoning denatured proteins for subsequent refolding [89]. Although under mild stress conditions protein unfolding is negligible, LD accumulation may reflect a metabolic change in preparation for fulfilling such a homeostatic function.

### Membrane rigidization occurs very rapidly in response to heat challenge

**Fig 8:**
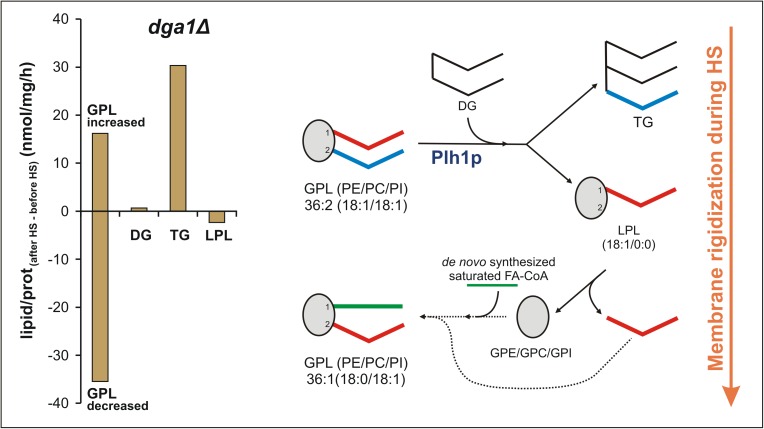
Proposed mechanism of membrane rigidization in response to HS for the *dga1Δ* strain. Net changes expressed as lipid/prot_(after HS–before HS)_ (nmol/mg/h) values (left; average data from n = 3 independent experiments are shown), and schematic representation of TG-supported membrane rigidization (right) in the *dga1Δ* strain. DG, diacylglycerol; GPC/GPE/GPI, phosphorylated headgroups; GPL, glycerophospholipid; LPL, lysophospholipid; PC, phosphatidylcholine; PE, phosphatidylethanolamine; PI, phosphatidylinositol; TG, triacylglycerol.

Concerning the kinetics and mechanism of HS-induced TG-supported membrane rigidization, we considered quantitative changes expressed as lipid/protein_(after HS–before HS)_ (nmol/mg/h) values. Based on our analysis of these data, we present a hypothesis in Fig 8 using the example of the *dga1Δ* strain, where TG can be produced exclusively from GPL substrates upon Plh1p activation. Because the products and substrates act in equimolar proportions (Fig 1), the sum of potential substrate species which were decreased (molecular species of PE, PC and PI ~35 nmol/mg prot/h; ~70% of which is due to decrease in 36:2 species) could potentially fulfil the FA requirement for TG formation (30 nmol/mg prot/h; Fig 8, left side). However, DG and LPL levels did not detectably change. Therefore, the amount of DG that would have to be consumed for TG synthesis (30 nmol/mg prot/h) was, in fact, completely supplemented either by *de novo* synthesis or by PLC/PLD action (S2 Fig). Of note, this is equivalent to four times the basal DG mass. Similarly, the amount of LPL produced (30 nmol/mg prot/h) was completely reused. Furthermore, in addition to GPL species that were reduced, there were GPL components (almost exclusively 36:1 and 34:1) that demonstrated considerable increases (16 nmol/mg prot/h; Fig 8, left side). We must also consider that, after removal of 18:1 from the preferred *sn2* position of the major 36:2 species by Plh1p [32], the major LPL product contains 18:1 in the *sn1* position (Fig 8, right side). Nevertheless, as confirmed by fragmentation experiments, the elevated membrane lipid species 36:1 and 34:1 contain 18:0 and 16:0 in the *sn1* positions, respectively. This means that a substantial portion of LPLs had to be first further catabolized to the corresponding phosphorylated headgroup (GPC/GPE/GPI) and then reacylated by two sequential steps in order to enable acyl chain exchange (Fig 8, right side) or subjected to *de novo* lipid synthesis. Saturated acyl chains for reacylation must be derived from *de novo* FA synthesis, while unsaturated acyl groups could be recycled after cleavage. It is conceivable that the same mechanism is also operational for the *plh1Δ* and WT strains (S5 Fig).

The above calculations suggest that at least 120 nmol lipid/mg protein underwent processing during the 1-hour HS (*dga1Δ* strain, S3 Table). This value is equivalent to ~65% of the total initial membrane lipid content. The net flux data calculated here represent the sum of several reversible and multicomponent reactions for both substrates and products (S2 Fig) and, therefore, likely underestimate the real dynamic alterations. Nevertheless, they suggest very rapid membrane reorganization in addition to the otherwise fairly intensive turnover of membrane lipids and post-synthetic deacylation/reacylation processes under normal physiological conditions [90–92].

### Conclusions

Organisms have developed several mechanisms to maintain proper membrane composition and physical state during HS. Although lipidomic studies have already characterized the lipidome of *S*. *cerevisiae* at elevated growth temperatures [54,93], to the best of our knowledge, this is the first detailed lipidomic study for the fission yeast *S*. *pombe* in the presence or absence of mild HS conditions (*i*.*e*., where growth is not enabled, but cells remain viable) and which demonstrates a functional link between membrane lipid adaptation and TG metabolism.

Storage lipid synthesis is non-essential in *S*. *cerevisiae* [65] and non-essential in the exponential growth phase at normal temperature for *S*. *pombe* ([32] and this study). However, the present study demonstrates that TG formation significantly influences the basal lipid composition and is important under HS conditions. The absence of TG in heat-stressed DKO cells was associated with severe growth retardation, which was paralleled by numerous measurable deficiencies in membrane lipid composition and enhanced signalling lipid generation. Consistent with this finding, it was suggested that LDs, which in yeast remain associated with the ER throughout their life cycle, may play an important role in membrane-and organelle-remodelling events [94].

It was earlier proposed that LD biogenesis during stress may represent a survival strategy, serving to divert structural phospholipids into energy-generating substrates [27]. Here we extrapolate further by suggesting that the production of TG in response to HS not only represents a passive mode for conversion of stored intracellular chemical energy, but that it actively contributes to the stress adaptation mechanism by decreasing unsaturated FAs (directly by Plh1p or indirectly by Dga1p) in structural lipids, enabling their replacement with *de novo* synthesized saturated FAs.

In summary, stress-induced TG synthesis (and the concomitant LD biogenesis) assists membrane adaptation, the synthesized LDs may fulfil holdase chaperone function during stress, while in the post-stress state they serve to meet energy demands and supply FAs for membrane resynthesis. TG synthesis is an energy consuming process, however, it may possibly represent a short-term cost that is expected to be repaid by a secured future survival benefit.

## Supporting information

S1 Appendix Validation of one-step MeOH extraction. (DOCX)

S2 Appendix Lipidomic analysis details. (DOCX)

S1 Table *S*. *pombe* strains used in this study. (DOCX)

S2 Table Detailed lipidomic dataset. (XLSX)

S3 Table Minimal lipid amounts processed during HS for *dga1Δ*. (DOCX)

S1 Fig Cell viability assessment. (DOCX)

S2 Fig Lipid metabolism in *S*. *pombe*. (DOCX)

S3 Fig Choline supplementation. (DOCX)

S4 Fig Changes in FA fluxes for individual FAs. (DOCX)

S5 Fig Changes in lipid fluxes for the WT and *plh1Δ* strains. (DOCX)
