# Supplementary material for: Metabolic crosstalk between membrane and storage lipids facilitates heat stress management in Schizosaccharomyces pombe
Source: PLoS One. 2017 Mar 10;12(3):e0173739. doi: 10.1371/journal.pone.0173739 (PMC5345867; doi:10.1371/journal.pone.0173739)
Supplement: S1 Appendix — (DOCX) [file pone.0173739.s001.docx]

**S1 Appendix. Validation of one-step MeOH extraction**

Validation of one-step MeOH extraction of lipids was performed on *S. pombe* and *S. cerevisiae* homogenates and compared to extracted lipids obtained by the gold-standard two-phase Folch extraction [1]. For this, 25 μL *S. pombe* or *S. cerevisiae* homogenate was extracted using 500 μL MeOH or subjected to standard Folch extraction. The latter was carried out with slight modifications according to [2]. Briefly, the homogenate volume was adjusted to 200 μL with water, 500 μL MeOH and 250 μL chloroform were added to form one phase (chloroform:methanol:water = 1:2:0.8, by vol.). The mixture was left at RT for 1 h with periodical vortexing, then 750 μL chloroform and 175 μL 0.2 M KCl were added to induce phase separation (final composition of chloroform:methanol:water = 2:1:0.75, by vol.). The lower chloroform phase was evaporated under vacuum and the residue redissolved in 500 μL chloroform:methanol (1:1, by vol.). In both cases, the extraction standard mix was dissolved in the MeOH component. The extraction standards, their amounts and recoveries are summarized in Table in S1 Appendix. With respect to lipid recoveries (for *S. pombe*), the MS measurements revealed the efficacy of MeOH extraction across the lipid species examined (PC, PE, PI, PS, PA, Cer, DG and TG).

In addition, the lipid class and lipid species composition of both *S. pombe* and *S. cerevisiae* extracts were determined and found to be comparable by the two extraction methods for GPLs, DG, TG and EE (Figure in S1 Appendix). For *S. pombe*, also the SLs Cer, IPC and MIPC were detected in very similar amounts by both extraction procedures. Of note, for *S. cerevisiae*, the more polar main structural sphingolipid MIP_2_C could be recovered only by the MeOH extraction method (Figure in S1 Appendix), while the levels of Cer and IPC were more enriched in the Folch extracts regardless of whether the extraction was carried out at RT or at 0 °C. This raises the possibility that the absence of MIP2C in the Folch extract is not due to its non-extractability, but is a result of some unusual *S. cerevisiae*-specific enzymatic degradation. Since the level of MIPC is very low in *S. cerevisiae* (<0.1% of GPLs), but represents a sizeable amount in *S. pombe* (2-3% of GPLs), we performed a mixed extraction with combined *S. pombe* and *S. cerevisiae* homogenates to find out whether MIPC becomes depleted by this putative enzymatic activity. However, the mixing results showed that the amount of MIPC contributed by *S. pombe* remained unchanged. We did not further seek to clarify the reason for this observation.

Importantly, unspecific lipid hydrolysis during homogenization/extraction can be excluded based on the absence of the corresponding lyso species or released free fatty acids from the glycerophospholipid extraction standards which should appear due to such phenomena.

The MeOH extraction has been reported to be applicable for higher organisms as well in literature reports [3] and in our prior experience for various cell cultures (B16, CHO, Hela, lymphocytes) and numerous tissue samples including liver, muscle, Drosophila testis [4], heart, brain and blood. From brain tissues the higher order gangliosides, which remain completely unrecovered by Folch extraction, were efficiently extracted, whereas from plasma, the recovery of the dominant neutral lipids (cholesteryl ester and TG) was as efficient as recovery using the Folch procedure.

The typical extract concentration was in the range of 2-10 mg tissue samples (wet weight), 2x10^6^ mammalian cells, 10^7^ lymphocytes or 10^8^ yeast cells/mL MeOH. The procedure allows at least ten-fold dilution of the extract before injection, whereby the final lipid concentration corresponds to the reported “shotgun range” (low pmol/μL, where a linear correlation of the absolute ion intensity with the concentration of each polar lipid class is obtained [5]). Although not tested in detail, we propose that greater starting material quantity would limit the efficiency of MeOH extraction for high fat-content samples due to incomplete extractability of neutral lipids. High salt-containing samples may be problematic due to increased ion suppression which unfavourably affects the sensitivity of MS measurements.

**Table in S1 Appendix. Lipid extraction recoveries**

| Lipid | Amount per extraction (nmol) | MeOH | Folch/KCl |
| --- | --- | --- | --- |
| PC (di20:0) | 2.36 | 94 ± 2 | 93 ± 2 |
| PE (diphythanoyl) | 1.25 | 98 ± 5 | 90 ± 6 |
| PI (di16:0) | 1.24 | 99 ± 3 | 99 ± 4 |
| PS (diphythanoyl) | 0.59 | 97 ± 5 | 80 ± 7 |
| PA (diphythanoyl) | 0.07 | 92 ± 6 | 75 ± 6 |
| Cer (35:1:2) | 0.09 | 100 ± 4 | 97 ± 2 |
| DG (di17:0) | 0.24 | 98 ± 6 | 100 ± 5 |
| TG (tri22:1) | 0.37 | 98 ± 4 | 100 ± 3 |

MS analysis details are given in the Materials and methods section of the main article and in S2 Appendix. Lipid recoveries were calculated by comparison with the corresponding internal standard quantities applied as in S2 Appendix taken the non-extracted extraction standard mix components as 100%. Values are expressed as mean ± SD (%), n = 4 independent experiments.


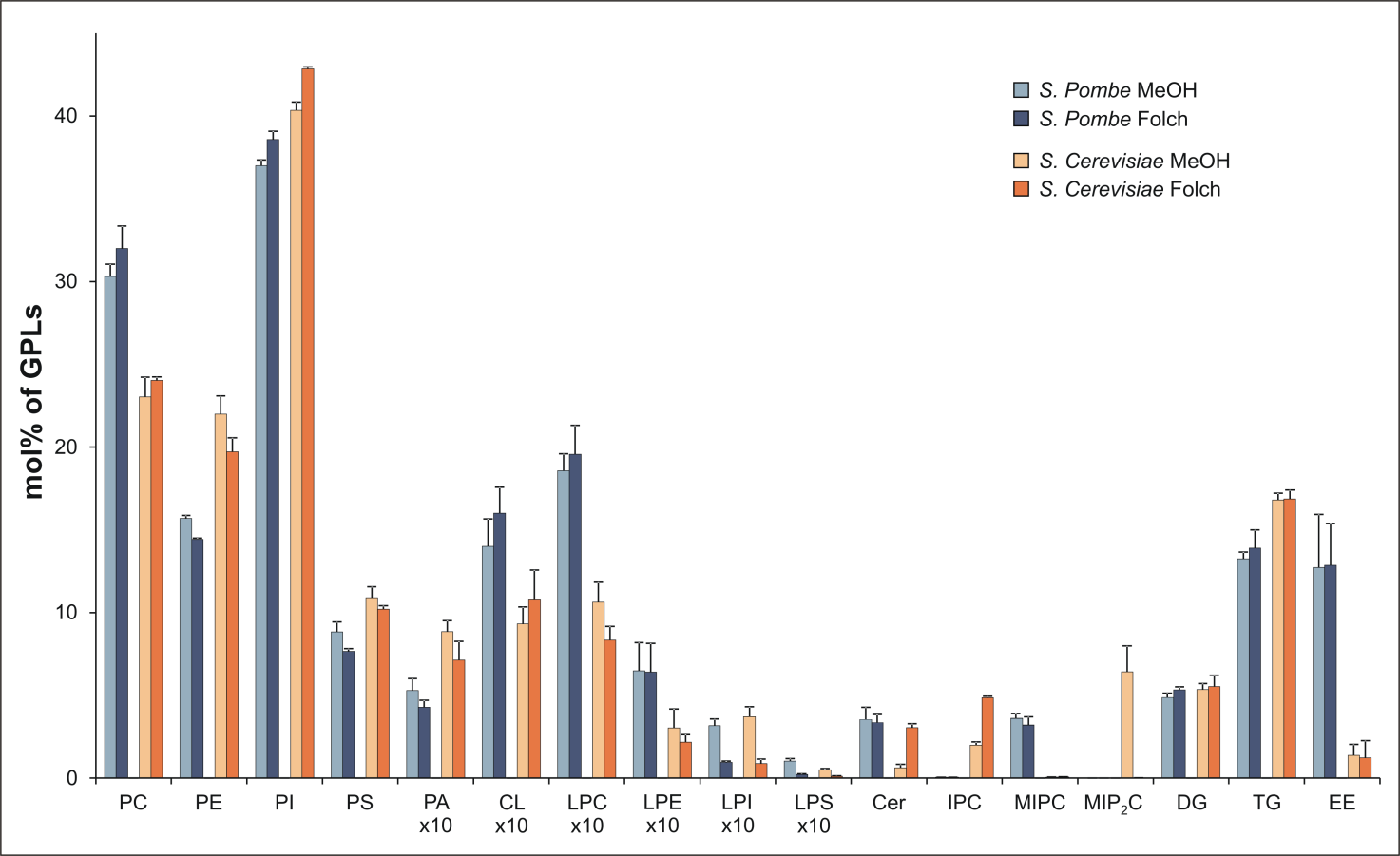


**Figure in S1 Appendix. Comparison of MeOH and Folch extractions.**

Values are expressed as mean ± SD (mol% of GPLs), n = 4. PC, phosphatidylcholine, PE, phosphatidylethanolamine, PI, phosphatidylinositol, PS, phosphatidylserine, PA, phosphatidic acid, CL, cardiolipin. LPC, LPE, LPI and LPS denote the corresponding lyso derivatives of PC, PE, PI and PS. Cer, ceramide, IPC, inositolphosphoceramide, MIPC, mannosyl-inositolphosphoceramide, MIP_2_C, mannosyl-diinositolphosphoceramide.

**References in S1 Appendix**

1. Folch J, Lees M, Sloane Stanley GH. A simple method for the isolation and purification of total lipides from animal tissues. J Biol Chem. 1957;226: 497–509. doi:LIPIDS/determination

2. Balogh G, Péter M, Liebisch G, Horváth I, Török Z, Nagy E, et al. Lipidomics reveals membrane lipid remodelling and release of potential lipid mediators during early stress responses in a murine melanoma cell line. Biochim Biophys Acta - Mol Cell Biol Lipids. 2010;1801: 1036–1047. doi:10.1016/j.bbalip.2010.04.011

3. Klem S, Klingler M, Demmelmair H, Koletzko B. Efficient and specific analysis of red blood cell glycerophospholipid fatty acid composition. PLoS One. 2012;7: e33874. doi:10.1371/journal.pone.0033874

4. Laurinyecz B, Péter M, Vedelek V, Kovács AL, Juhász G, Maróy P, et al. Reduced expression of CDP-DAG synthase changes lipid composition and leads to male sterility in Drosophila. Open Biol. 2016;6: 50169. doi:10.1098/rsob.150169

5. Han X, Yang K, Gross RW. Multi-dimensional mass spectrometry-based shotgun lipidomics and novel strategies for lipidomic analyses. Mass Spectrometry Reviews. 2012. pp. 134–178.
