# Supplementary material for: Metabolic crosstalk between membrane and storage lipids facilitates heat stress management in Schizosaccharomyces pombe
Source: PLoS One. 2017 Mar 10;12(3):e0173739. doi: 10.1371/journal.pone.0173739 (PMC5345867; doi:10.1371/journal.pone.0173739)
Supplement: S2 Appendix — (DOCX) [file pone.0173739.s002.docx]

**S2 Appendix. Lipidomic analysis details**

For quantification, 15 μL lipid extract was diluted with 285 μL infusion solvent mixture (chloroform:methanol:iso-propanol 1:2:1, by vol.) containing an internal standard mix. The ion formats for quantification and the internal standard spike amounts are listed in Table in S2 Appendix. Next, the mixture was halved, and 5% dimethylformamide (additive for the negative ion mode) or 3 mM ammonium chloride (additive for the positive ion mode) were added to the split sample halves. Each of the quantified lipid species accounted for more than 0.5% within its lipid class. Under the applied MS conditions we did not observe in-source fragmentation yielding either PA (from PS), lyso GPL species (from the parent GPL) or DMPE (from PC).

For fragmentation analyses, HCD collision energy values were detemined for each lipid class. Data-dependent MS/MS fragmentation experiments were performed based on mass lists from survey scans. Data files generated by LipidXplorer queries were further processed by in-house Excel macros.

FFAs were quantitated by using a gas chromatography (GC)-MS system after thin layer chromatography separation of major lipid classes as described in [1]. Noteworthy, the quantitative data obtained for total polar lipids (GPL + SL), DG, TG and EE were highly similar by the two different MS techniques (*i.e.,* FA determination using GC-MS and ESI-MS). These results validate the reliability of our ESI-MS method.

**Table in S2 Appendix. MS quantitation details**

| Lipid Class | Ion format | Internal standard (IS) | IS amount in infusion solvent mix (pmol) |
| --- | --- | --- | --- |
| PC, LPC | [M+H]^+^ | PC D31-16:0/18:1 | 76 |
| PE, MMPE, DMPE, LPE | [M−H]^−^ | PE D31-16:0/18:1 | 53 |
| PI, LPI, IPC, MIPC | [M−H]^−^ | PI D31-16:0/18:1 | 46 |
| PS, LPS | [M−H]^−^ | PS D31-16:0/18:1 | 25 |
| PG | [M−H]^−^ | PG D31-16:0/18:1 | 1 |
| PA | [M−H]^−^ | PA D31-16:0/18:1 | 6 |
| CL | [M−2H]^2−^ | CL tetra14:0 | 1 |
| Cer | [M+Cl]^−^ | Cer t18:0/16:0 | 5 |
| DG | [M+NH_4_]^+^ | DG di22:1 | 7 |
| TG | [M+NH_4_]^+^ | TG tri17:1 | 9 |
| EE | [M+NH_4_]^+^ | CE 18:1 | 2 |

**Reference**

1. Balogh G, Péter M, Liebisch G, Horváth I, Török Z, Nagy E, et al. Lipidomics reveals membrane lipid remodelling and release of potential lipid mediators during early stress responses in a murine melanoma cell line. Biochim Biophys Acta - Mol Cell Biol Lipids. 2010;1801: 1036–1047. doi:10.1016/j.bbalip.2010.04.011
