# Supplementary material for: Metabolic crosstalk between membrane and storage lipids facilitates heat stress management in Schizosaccharomyces pombe
Source: PLoS One. 2017 Mar 10;12(3):e0173739. doi: 10.1371/journal.pone.0173739 (PMC5345867; doi:10.1371/journal.pone.0173739)
Supplement: S1 Table — (DOCX) [file pone.0173739.s003.docx]

**S1 Table. *S. pombe* strains used in this study**

| Name | Genotype | Source/Reference |
| --- | --- | --- |
| BRC1 | h^−^; leu1-32, ura4-D18 | (6) |
| BRC2 | h^+^; leu1-32, ura4-D18 | (6) |
| BRC41 | h^−^; leu1-32, ura4-D18; dga1::ura4^+^ | This study |
| BRC42 | h^−^; leu1-32, ura4-D18; plh1::ura4^+^ | This study |
| BRC46 | h^+^; leu1-32, ura4-D18; dga1::ura4^+^ | BRC2XBRC41 |
| BRC48 | h^−^; leu1-32, ura4-D18; dga1::ura4^+^; plh1::ura4^+^ | BRC42XBRC46 |

| *dga1Δ* deletion primers (5' - 3') | |
| --- | --- |
| w | CCAATATCTGACTGTCAATGA |
| x | CAATTCGCCCTATAGTGAGTCGTATCCCGTTCTATATAATCGTG |
| y | TCCTGTGTGAAATTGTTATCCGCTGGCCTATGCAATATGTTGTG |
| z | GCCTTCGATTTAATACTCCC |

| *plh1Δ* deletion primers (5' - 3') | |
| --- | --- |
| w | TGTCATCATCCTCTCAGGC |
| x | CAATTCGCCCTATAGTGAGTCGTAATATGAATTGCTTGAGCAGC |
| y | TCCTGTGTGAAATTGTTATCCGCTCTATCATTCAACCAATCATG |
| z | TGGCTAGTGGAACTTTCACC |
