# Supplementary material for: Metabolic crosstalk between membrane and storage lipids facilitates heat stress management in Schizosaccharomyces pombe
Source: PLoS One. 2017 Mar 10;12(3):e0173739. doi: 10.1371/journal.pone.0173739 (PMC5345867; doi:10.1371/journal.pone.0173739)
Supplement: S3 Table — (DOCX) [file pone.0173739.s005.docx]

**S3 Table. Minimal lipid amounts processed during HS for *dga1Δ***

| Process | Lipid class | Processed lipid (nmol/prot mg/h) |
| --- | --- | --- |
| Necessarily consumed for TG formation | GPL | 30 |
|  | DG | 30 |
| (Re)synthesized | DG | 30 |
| Degraded/Secreted/Used for new GPL production | LPL | 30 |
| ***SUM*** |  | ***120*** |
