# Supplementary material for: Metabolic crosstalk between membrane and storage lipids facilitates heat stress management in Schizosaccharomyces pombe
Source: PLoS One. 2017 Mar 10;12(3):e0173739. doi: 10.1371/journal.pone.0173739 (PMC5345867; doi:10.1371/journal.pone.0173739)
Supplement: S1 Fig — (DOCX) [file pone.0173739.s006.docx]

**
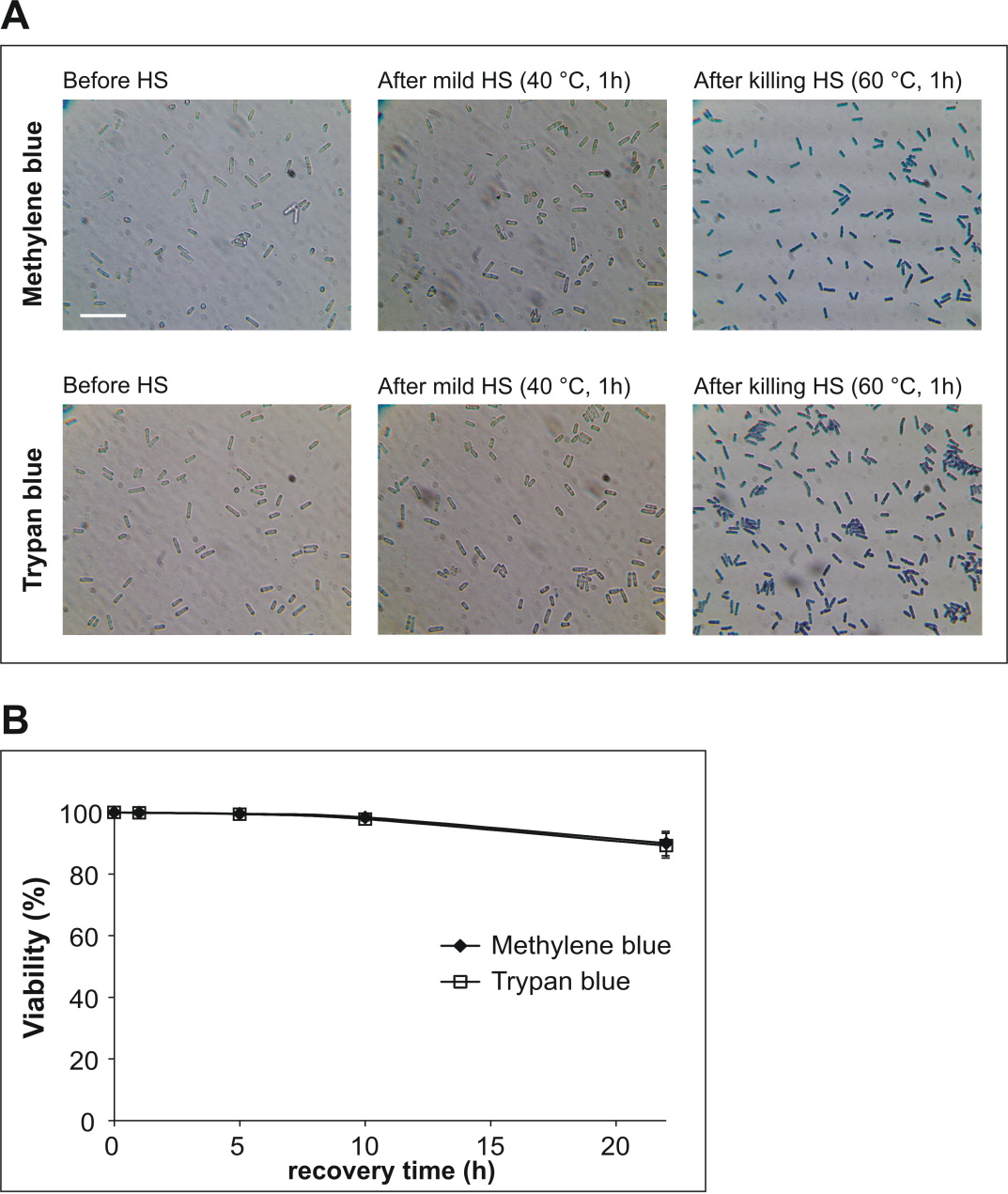
**

**S1 Fig. Cell viability assessment.**

Both methylene and trypan blue staining revealed that (A) DKO cells are quantitatively viable immediately after HS and (B) viablity remains at ≥90% during the lag phase. Cells were exposed to HS at 40 °C for 1 h. For (A), representative images of 5 independent experiments are shown; scale bar 20 µm; as a reference, heat-killed cells are also presented. For (B), cells were allowed to recover at 30 °C; viability values are expressed as mean ± SD; at least 300 cells were counted by microscopic observation for 3 independent experiments.
