# Supplementary material for: Metabolic crosstalk between membrane and storage lipids facilitates heat stress management in Schizosaccharomyces pombe
Source: PLoS One. 2017 Mar 10;12(3):e0173739. doi: 10.1371/journal.pone.0173739 (PMC5345867; doi:10.1371/journal.pone.0173739)
Supplement: S2 Fig — (DOCX) [file pone.0173739.s007.docx]

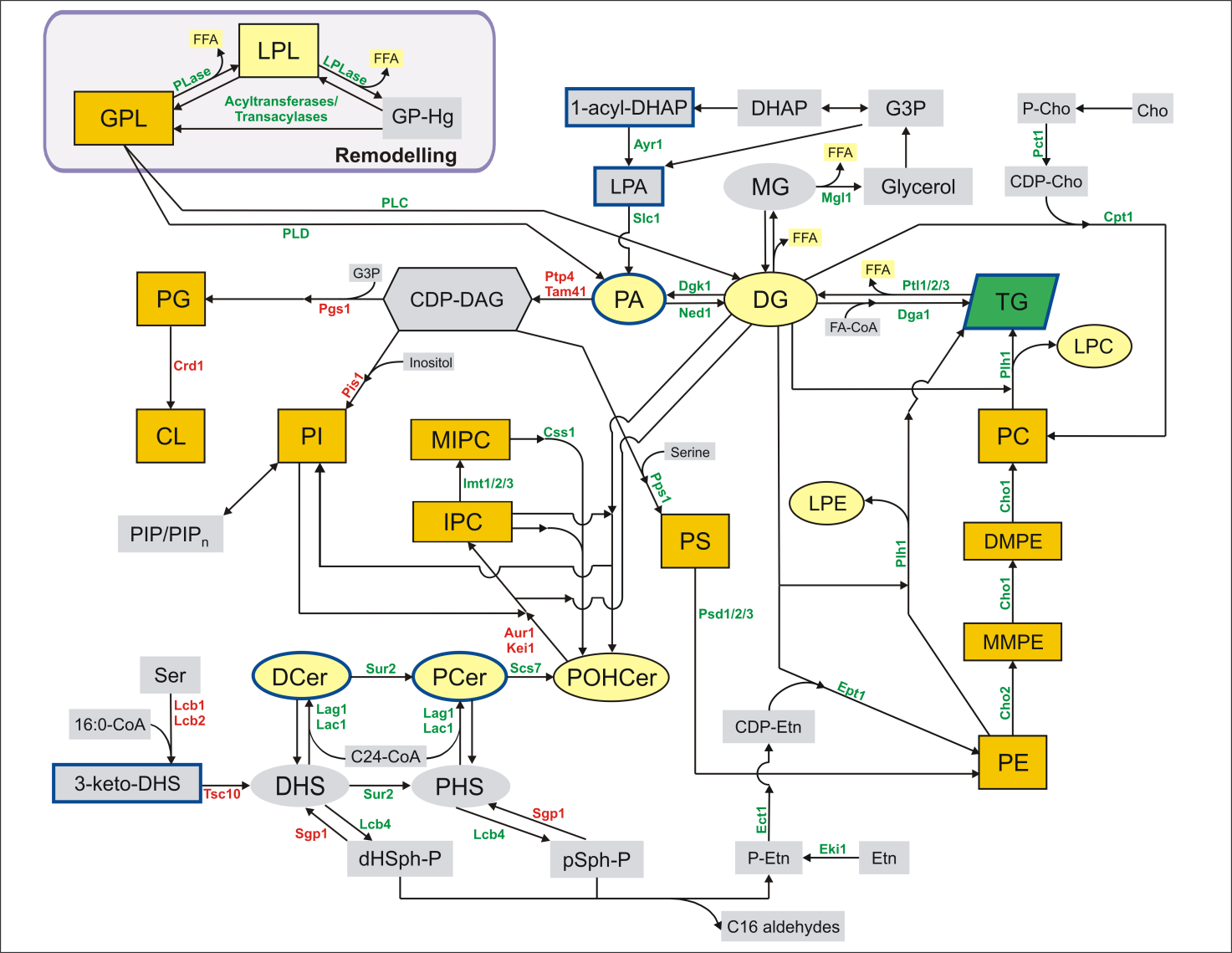


**S2 Fig. Lipid metabolism in *S. pombe*.**

Enzymes: Red – essential (predicted or assigned), Green - non-essential (predicted or assigned, based on PomBase.org). Lipids: orange - measured, structural; yellow - measured, signalling; green - measured, storage; grey - not measured. Blue frames indicate those lipids whose syntheses require FA-CoA. CL, cardiolipin; CDP-DG, cytidine diphosphate diacylglycerol; CDP-Etn, cytidine diphosphate ethanolamine; CDP-Cho, cytidine diphosphate choline; DCer/PCer/POHCer dihydro/phyto/hydroxy-phytoceramide; DG, diacylglycerol; DHS/PHS dihydro/phytosphingosine; DMPE, dimethyl-phosphatidylethanolamine; FFA, free fatty acid; GP-Hg, glycerophosphorylated headgroup (e.g. choline, ethanolamine, etc.); GPL, glycerphospholipid; IPC, inositolphosphoceramide; LPA, lysophosphatidic acid; LPC, lysophosphatidylcholine; LPE, lysophosphatidylethanolamine; LPL, lysophospholipid; MIPC, mannosyl-inositolphosphoceramide; MMPE, monomethyl-phosphatidylethanolamine; PA, phosphatidic acid; PC, phosphatidylcholine; PE, phosphatidylethanolamine; PG, phosphatidylglycerol; PI, phosphatidylinositol; PIP/PIP_n_, phosphatidylinositol phosphates; PS, phosphatidylserine; TG, triacylglycerol.
