# Supplementary material for: Metabolic crosstalk between membrane and storage lipids facilitates heat stress management in Schizosaccharomyces pombe
Source: PLoS One. 2017 Mar 10;12(3):e0173739. doi: 10.1371/journal.pone.0173739 (PMC5345867; doi:10.1371/journal.pone.0173739)
Supplement: S3 Fig — (DOCX) [file pone.0173739.s008.docx]

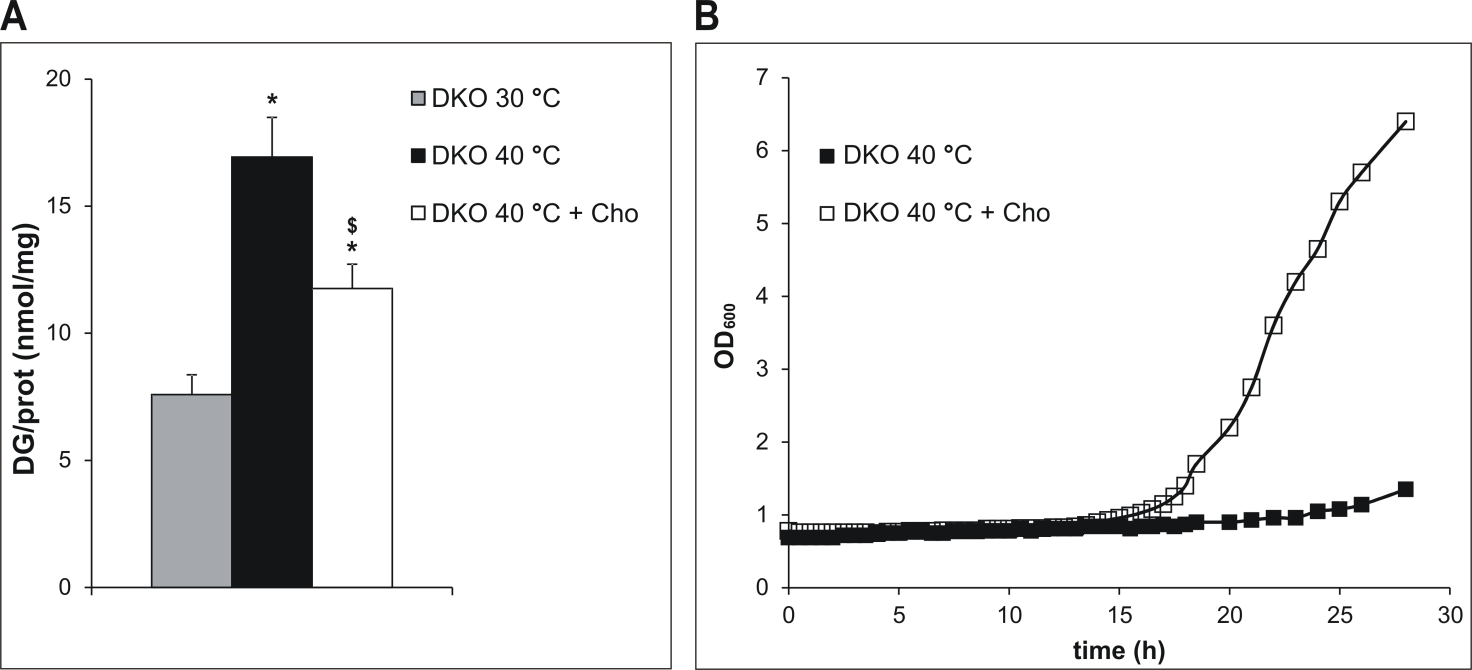


**S3 Fig. Choline supplementation.**

Choline (Cho) supplementation (A) significantly reduced the extent of DG increase, and (B) essentially shortened the growth retardation of heat-stressed DKO cells. *S. pombe* DKO cells were exposed to HS at 40 °C for 1 h and supplemented with or without 1 mM choline. Samples were collected for lipidomic analysis immediately after HS (for A; values are expressed as mean ± SD, n = 3; * *p*˂0.001 (vs 30 °C), $ *p*<0.001 (vs 40 °C)), or were allowed to recover at 30 °C, and growth curves were recorded (for B; representative images of 3 independent experiments are shown).
