# Supplementary material for: Metabolic crosstalk between membrane and storage lipids facilitates heat stress management in Schizosaccharomyces pombe
Source: PLoS One. 2017 Mar 10;12(3):e0173739. doi: 10.1371/journal.pone.0173739 (PMC5345867; doi:10.1371/journal.pone.0173739)
Supplement: S4 Fig — (DOCX) [file pone.0173739.s009.docx]

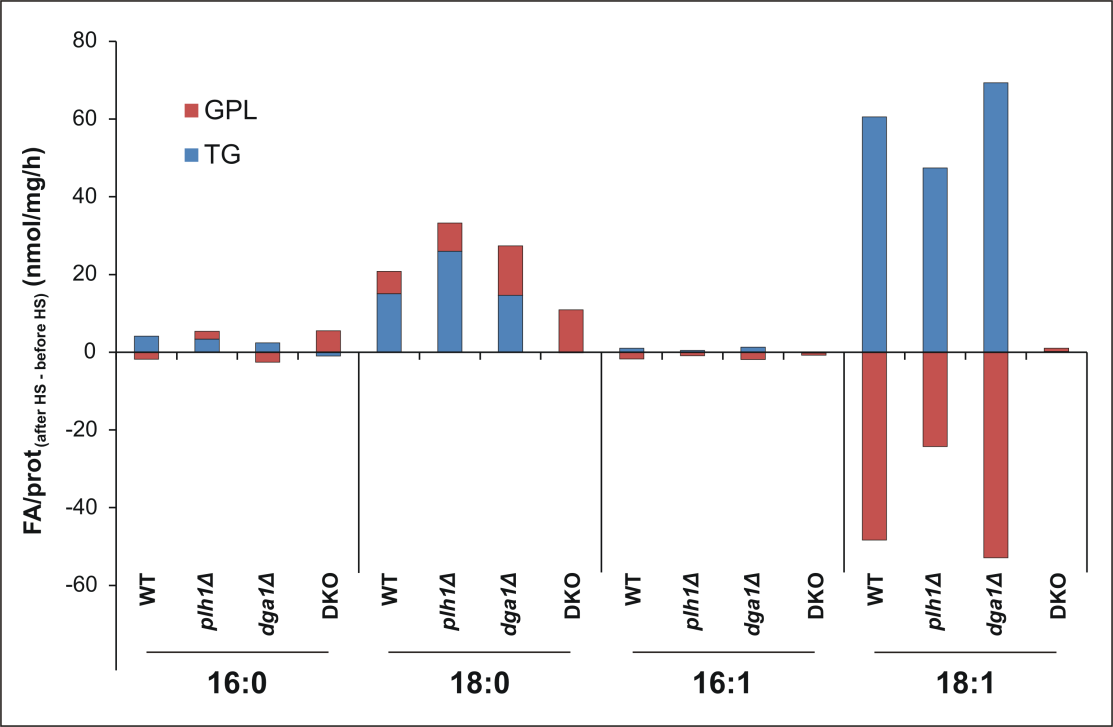


**S4 Fig. Changes in FA fluxes for individual FAs.**

Data are expressed as FA/prot_(after HS – before HS)_ (nmol/mg/h) values for long chain saturated (16:0 and 18:0) and unsaturated (16:1 and 18:1) FAs in the GPL and TG fractions. Average values are shown from n = 3 (for *plh1Δ* and *dga1Δ*), n = 4 (for DKO), and n = 7 (for WT) independent experiments. Data were reconstituted based on ESI-MS/MS fragmentation results.
