# Supplementary material for: Metabolic crosstalk between membrane and storage lipids facilitates heat stress management in Schizosaccharomyces pombe
Source: PLoS One. 2017 Mar 10;12(3):e0173739. doi: 10.1371/journal.pone.0173739 (PMC5345867; doi:10.1371/journal.pone.0173739)
Supplement: S5 Fig — (DOCX) [file pone.0173739.s010.docx]

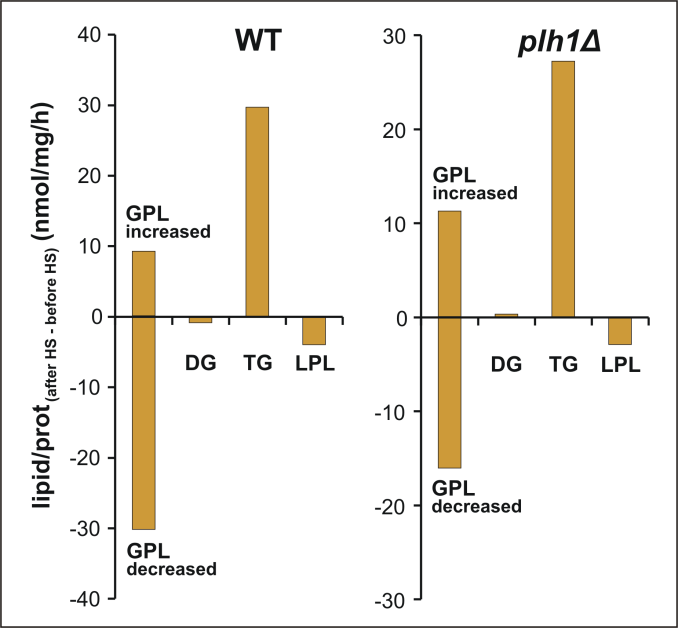


**S5 Fig. Changes in lipid fluxes for the WT and *plh1Δ* strains.**

Net changes are expressed as lipid/prot_(after HS – before HS)_ (nmol/mg/h) values. Average data are shown from n = 7 (for WT) and n = 3 (for *plh1Δ*) independent experiments.
